# Quality of life, salivary cortisol and atopic diseases in young children

**DOI:** 10.1371/journal.pone.0214040

**Published:** 2019-08-30

**Authors:** Leif Bjarte Rolfsjord, Håvard Ove Skjerven, Egil Bakkeheim, Teresa Løvold Berents, Kai-Håkon Carlsen, Karin C. Lødrup Carlsen

**Affiliations:** 1 Department of Paediatrics, Innlandet Hospital Trust, Elverum, Norway; 2 Division of Paediatrics and Adolescent Medicine, Oslo University Hospital, Oslo, Norway; 3 Institute of Clinical Medicine, University of Oslo, Oslo, Norway; 4 Department of Dermatology, Oslo University Hospital, Oslo, Norway; Norwegian Institute of Public Health, NORWAY

## Abstract

**Background:**

Children with atopic disease may have reduced health-related quality of life (QoL) and morning cortisol. Possible links between QoL, morning cortisol and atopic disease are unclear. We aimed to determine if QoL was associated with morning salivary cortisol at two years of age, and if asthma, atopic dermatitis and/or allergic sensitisation influenced this association. Secondarily, we aimed to determine if QoL at one year of age was associated with salivary cortisol one year later.

**Methods and findings:**

The Bronchiolitis All SE-Norway study included infants during hospitalisation for acute bronchiolitis in infancy (bronchiolitis group) and population-based control infants (controls). The present study included all 358 subjects with available Infant Toddler Quality of Life Questionnaire (ITQOL) from parents, consisting of 13 domains and morning salivary cortisol at two years of age. Answers from the same 0–100 score questionnaire, with optimal score 100 nine months after enrolment, was also available for 289 of these children at about one year of age. Recurrent bronchial obstruction was used as an asthma proxy. Atopic dermatitis was defined by Hanifin and Rajka criteria and allergic sensitisation by a positive skin prick test. Due to different inclusion criteria, we tested possible interactions with affiliation groups. Associations between QoL and cortisol were analysed by multivariate analyses, stratified by bronchiolitis and control groups due to interaction from affiliation grouping on results. At two years of age, QoL decreased significantly with decreasing cortisol in 8/13 QoL domains in the bronchiolitis group, but only with General health in the controls. The associations in the bronchiolitis group showed 0.06–0.19 percentage points changes per nmol/L cortisol for each of the eight domains (p-values 0.0001–0.034). The associations remained significant but diminished by independently including recurrent bronchial obstruction and atopic dermatitis, but remained unchanged by allergic sensitisation. In the bronchiolitis group only, 7/13 age and gender adjusted QoL domains in one-year old children were lower with lower cortisol levels at two years of age (p = 0.0005–0.04).

**Conclusions:**

At two years, most QoL domains decreased with lower salivary cortisol among children who had been hospitalised for acute bronchiolitis in infancy, but for one domain only among controls. Recurrent bronchial obstruction and to a lesser extent atopic dermatitis, weakened these associations that nevertheless remained significant. After bronchiolitis, lower QoL in one-year old children was associated with lower salivary cortisol at two years.

## Introduction

Development of asthma has been associated with acute bronchiolitis [1, 2] and asthma with reduced basal morning salivary cortisol, also in children without current use of inhaled corticosteroids [3]. Asthma [4, 5], atopic dermatitis [6], previous hospitalisation for acute bronchiolitis [7, 8], and psychological and physical stress [9, 10] have been associated with reduced health-related quality of life (QoL). Subjects exposed to pre- or postnatal stress may have a lower cortisol after exposure to acute stress if atopic as opposed to non-atopic subjects with the same exposures tending to have higher cortisol [11].

The generic, parent-based Infant Toddler Quality of Life Questionnaire (ITQOL-97) has shown reduced QoL in young children with obstructive airways disease [12], AD [8] and some other diseases [13]. Only five of the concepts or domains of ITQOL-97 specify a time period, each of past four weeks. In comparison, in children, chronic cough is increasingly defined as having lasted for more than four weeks ([14]. Thus, ITQOL-97 may possibly be sensitive to chronic disease and stress.

In the Bronchiolitis All SE-Norway study, we collected extensive information about the participants, including history and signs of atopic disease [15–17]. In the present add-on exploratory study, we applied the same survey of parent-reported QoL of the children and concepts of impact on the parents, i.e. ITQOL-97, at two time points. Based on published studies on morning or acute stress-induced cortisol [18–20], we hypothesised that *low* cortisol levels in periods without illness requiring acute hospitalisation may contribute to development of asthma. We further hypothesised that reduced QoL some months after severe acute illness in early life may be a marker of chronic stress, with subsequent lower future salivary cortisol levels.

We therefore primarily aimed to determine if QoL was associated with morning salivary cortisol at two years of age, and if asthma, atopic dermatitis and/or allergic sensitisation modified this association. Secondarily, we aimed to determine if QoL at one year of age was associated with salivary cortisol at two years.

## Materials and methods

### Study design

**Fig 1:**
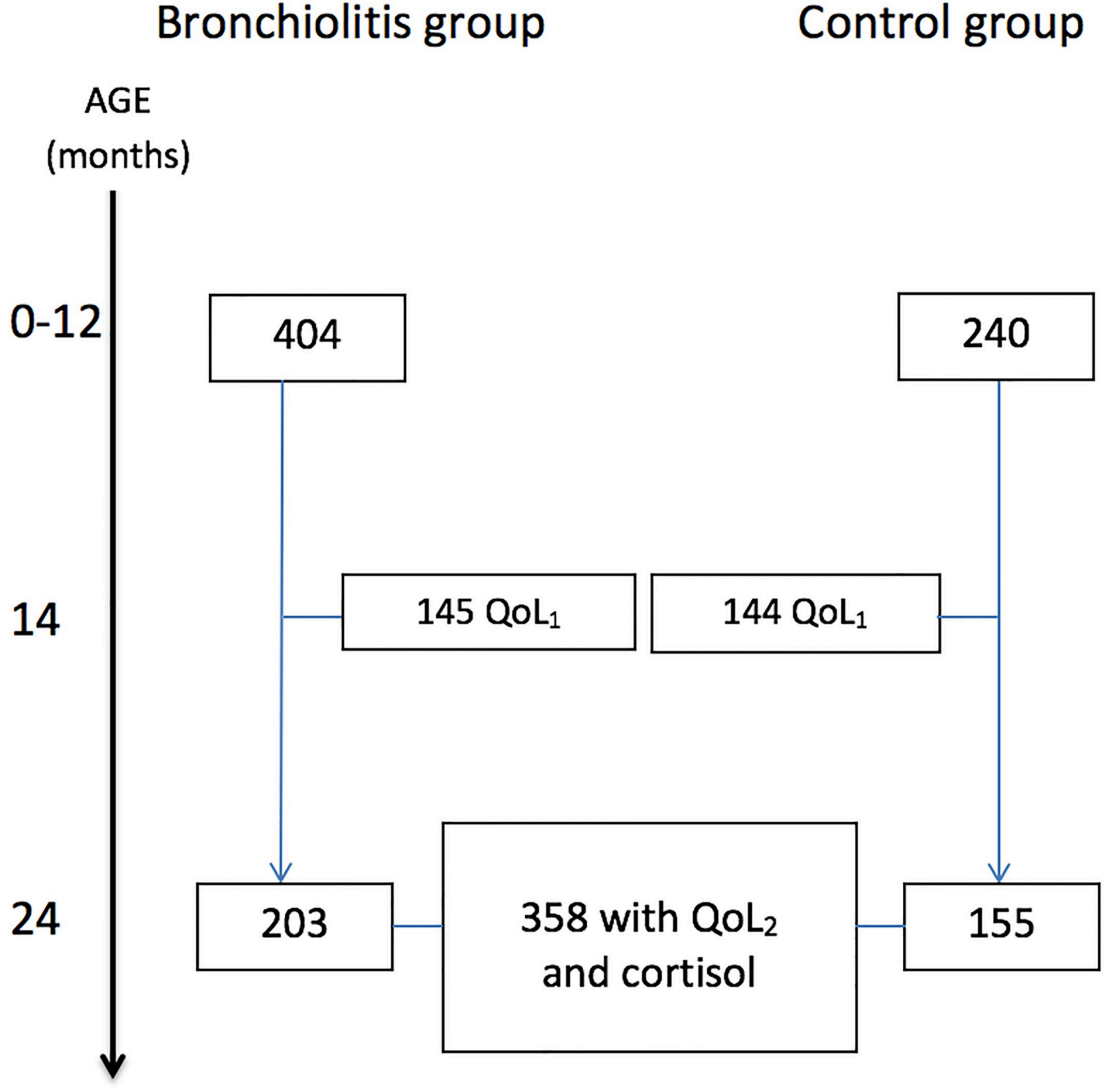
The Fig outlines the number of infants enrolled in the Bronchiolitis All SE-Norway study (top, n = 644) who were subsequently included in the present study (n = 358) for analyses based upon available Quality of life (QoL) and/or salivary morning cortisol at 24 months of age. The QoL questionnaires were completed nine months after enrolment at approximately 14 months of age (QoL_1_) as well as at the time of the clinical examination at 24 months of age (QoL_2_).

From the source population of 644 children included in the Bronchiolitis ALL SE-Norway study enrolling infants who were hospitalised for acute bronchiolitis and controls recruited from a general population [8], we included all 358 children with available salivary cortisol and QoL at 24 months of age. The bronchiolitis group consisted of 203 infants with moderate to severe acute bronchiolitis at inclusion, and 155 were controls. For details, see Fig 1 and Supporting Information.

Investigations at enrolment and at two years of age included clinical assessment, structured parental interviews and morning salivary sampling for cortisol. Skin prick test (SPT) for common inhalant and food allergens was performed at two years. Quality of life questionnaires were completed by parents nine months after enrolment (QoL_1_) [8, 21] and at two years of age (QoL_2_).

Caregivers of all children signed the informed written consents prior to study enrolment. The study was approved by the Regional Committee for Medical and Health Research Ethics and The Norwegian Data Protection Authority and registered in the Norwegian bio bank registry. The randomised clinical trial part of the study was registered in Clinical Trials.gov, no. NCT00817466 [16].

### Subjects

The mean (range) age of the 358 children in the present study was 5.2 (0.2–13.4) months at enrolment and 24.2 (17.6–34.7) months at the two-year investigation. The children in the bronchiolitis group compared to controls were shorter, more often exposed to second-hand smoke at inclusion and their parents had lower income, lower educational attainment and less often allergic rhinitis or AD (Table 1).

**Table 1:** Characteristics and asthma risk factors of the children at two years of age. All data are given as n and %, unless otherwise stated. The control group is the group from the reference population.

	Bronchiolitis group N = 203	Control group N = 155
Boys n (%)	117 (57.6%)	89 (57.4%)
Age months, mean (SD)	24.2 (3.2)	24.3 (3.7)
Weight kg, mean (SD)	13.2 (1.6)	12.9 (1.5)
Length cm, mean (SD)	87.0 *** (4.1)	88.7(4.2)
Breastfeeding at enrolment n (%)	149 (73.4%)	122 (78.7%)
Second-hand smoke exposure in infancy n (%)	25 (14.4%)**	5 (3.3%)
Second-hand smoke exposure at 2 years	5 (2.5%)	1 (0.7%)
Atopic manifestations defined at 2 years		
At least one n (%)	103 (50.7%)	37 (23.9%)
Two or more n (%)	19 (9.4%)	25 (7.3%)
rBO (at least 3 wheeze episodes) n (%)	98 (48.3%)***	22 (14.2%)
Atopic dermatitis at 2 years n (%)	30 (14.8%)	25 (16.1%)
Allergic sensitisation^1^ n (%)	17 (8.4%)	11 (7.4%)
At least one parent asthma n (%)	35 (22.2%)	46 (29.7%)
At least one parent AD n (%)	33 (18.2%)*	46 (29.7%)
At least one parent allergic rhinoconjunctivitis n (%)	62 (34.4%)***	84 (54.2%)
Higher education mothers^2^ n (%)	122 (70.1%)***	142 (91.6%)
Higher education fathers^2^ n (%)	100 (57.8%)***	129 (83.8%)
Income mothers^3^, mean (SD)	1.92 **	2.13
Income fathers^3^, mean (SD)	2.32 ***	2.59
Caucasian mother n (%)	189 (93.6%)	147 (94.8%)
Caucasian father n (%)	191 (95.0%)	143 (92.3%)

^1^Allergic sensitisation was defined by at least one positive skin prick test to common inhalant and food allergens

^2^Higher education at least three years after secondary school

^3^Annual income before tax. 1: <300.000 NOK. 2: 300.000–500.000 NOK. 3: >500.000 NOK.

* p<0.05

** p<0.01

***<0.001

### Methods

Atopic manifestations determined at two years of age, consisted of recurrent bronchial obstruction (rBO) as a proxy for asthma, atopic dermatitis and allergic sensitisation.

#### RBO

RBO was defined as at least three parentally reported episodes of wheeze at two years of age, in line with previous reports [15], with acute bronchiolitis included as one episode.

#### Atopic dermatitis

Atopic dermatitis was defined based upon the modified Hanifin and Rajka’s criteria (yes or no) [22], and severity by the SCORing Atopic Dermatitis index (SCORAD) (see Supporting Information for details).

#### Allergic sensitisation

Allergic sensitisation determined by SPT using 17 common inhalant and food allergens with Soluprick SQ allergen extracts, ALK, Hørsholm, Denmark, was defined as positive with at least one mean wheal diameter at least 3 mm greater than the negative control. Further details are given in the Supporting Information.

#### Morning salivary cortisol

Morning salivary cortisol was sampled by the parents on the first morning after enrolment in the bronchiolitis group, otherwise at home and brought to the investigation centre. Two Sorbette hydrocellulose microsponges were applied in the child’s mouth as soon as possible after their child’s first awakening after 6:00 a.m., before the first meal, and placed in appropriate prepared containers, as described elsewhere [23]. The samples were stored at -86°C and later analysed at Karolinska Institutet, Stockholm, with radioimmunoassay with monoclonal rabbit antibodies Codolet, France).

#### The Infant Toddler Quality of Life Questionnaire

The Infant Toddler Qualilty of Life Questionnaire (ITQOL-97) [12] completed by the parents included 97 questions within 13 domains scored from 0 (worst) to 100 (best), with no overall score. Accordingly, a change in QoL score is equivalent to the percentage point score change. The Overall health domain consisted of only one item: Is your child’s health excellent, very good, good, fair or poor? In line with others [24] and as previously reported [8, 21], with permission from the copyright holder, we recoded the domain Change in health from the original scores from 1–5 to 0–100 (zero meaning worst deterioration of health from one year ago, 50 meaning no change). Four domains (Change in health, General behaviour, Overall behaviour and Getting along) were recorded in children older than 12 months only [8]. The same questionnaire was filled in at both time points. For detailed information, see the attachment: ITQOL Survey Profile, provided by the trademark holder. There are 10 infant/ toddler focused concepts or domains. Behaviour is divided into two separately scored domains; Overall behaviour (1 item) and General behaviour (15 items). As there is no overall score, ITQOL-97 can be regarded as several surveys at a time, including three parent-focused items.

#### Outcomes and explanatory variables

The main outcome for our primary aim, QoL_2_, was reported by quantitative values per domain, and secondarily by the number of domains with significantly reduced QoL_2_ scores.

The main explanatory variables for the primary aim were morning salivary cortisol, and the three atopic manifestations rBO, AD and allergic sensitisation at two years of age. Further analyses reported in Supporting Information substituted the respective atopic manifestations by quantitative measures, i.e. the total number of wheeze episodes, the AD severity score SCORAD and the sum of SPT wheal diameters for influence on the associations between morning salivary cortisol and QoL_2_.

The main outcome of the secondary aim was morning salivary cortisol, with QoL_1_ as the explanatory factor.

### Statistical analysis

The bronchiolitis and control groups were compared by Pearson’s chi-square tests for categorical data and Student’s T- test for normally distributed numerical data, and otherwise with Welch test, unless otherwise stated.

**Fig 2:**
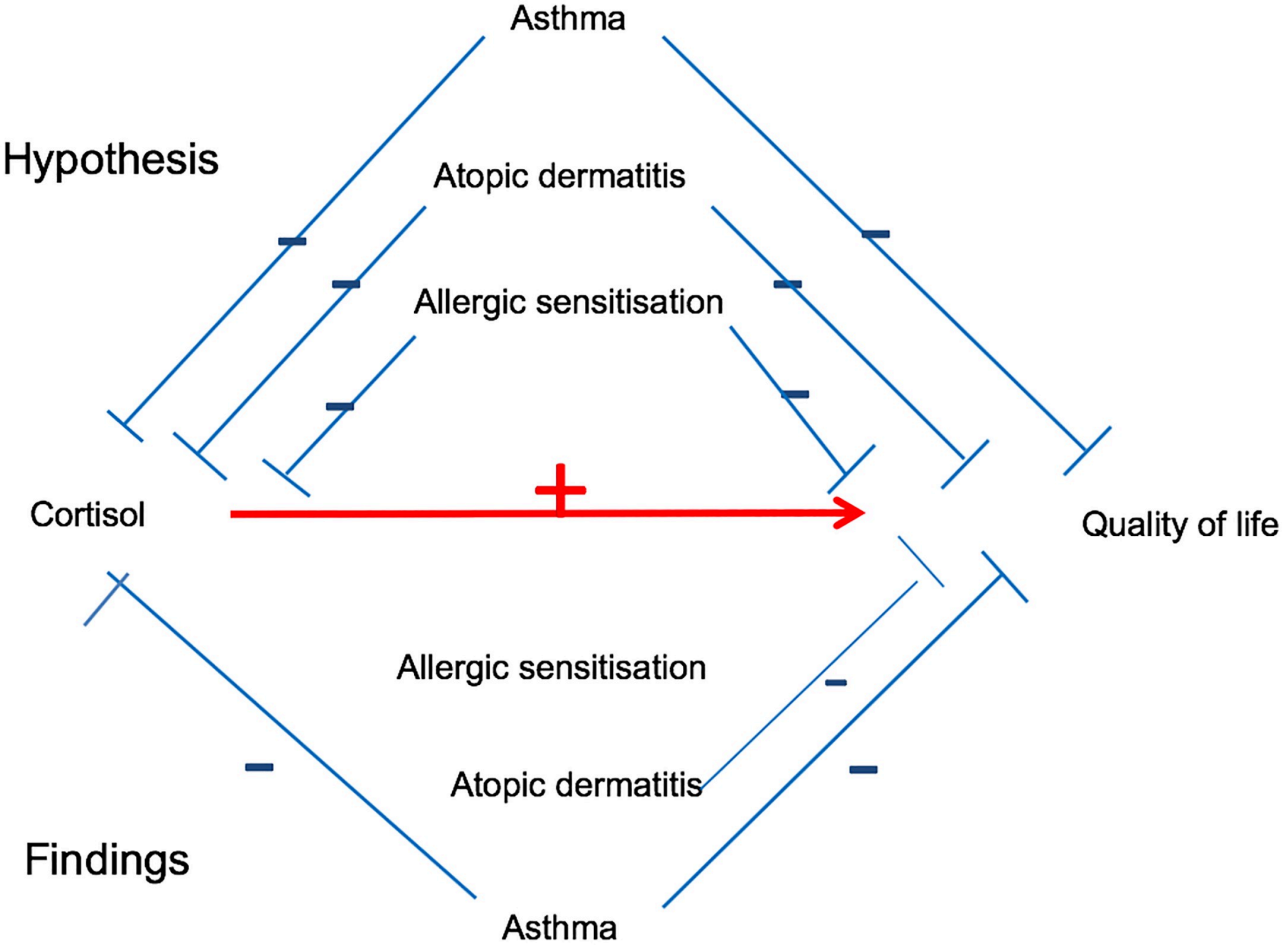
Directed acyclic graph showing hypothesised influence on cortisol and QoL_2_ by allergic diseases above the red line, and observed influence in the bronchiolitis group below the red line. The red line indicates the net result from the influence of allergic disease on the association between morning salivary cortisol and QoL_2_.

Due to non-normality of results and residuals, we used linear robust regression by Huber’s M method [25], for analyses including QoL and cortisol. Each atopic manifestation was included in robust regression models to assess their potential influence on both cortisol and QoL_2_, as well as the associations between the two (see Fig 2, hypothesis). To estimate the relative influence by rBO, AD and allergic sensitisation on QoL_2_, we calculated the percent change of the difference in score for each QoL domain, given per nmol/L change in cortisol. For comparison, we calculated the difference in each QoL domain score that was attributed to a difference in salivary cortisol level of 95th versus 5^th^ percentile (QoL score at the salivary cortisol level of 95^th^ percentile minus QoL score at the 5^th^ percentile). Salivary cortisol was studied as a continuous variable, and presented graphically by quartiles.

For graphical presentations of QoL versus cortisol levels and cortisol levels versus atopic manifestations we used data unadjusted for age and gender. In line with previously demonstrated associations between morning salivary cortisol and age as well as gender [23], we decided a priori to analyse age and gender adjusted associations between cortisol and QoL as well between QoL and atopic manifestations. The atopic manifestations were not considered to be possible confounders, as they could be causally associated with both cortisol and QoL_2_.

Using QoL_2_ as dependent variable in two-way regression analyses, we tested for interactions between the group affiliation (bronchiolitis or controls) and cortisol, as well as between atopic manifestations and cortisol. Due to interactions between group affiliation and salivary cortisol as well as AD, analyses were stratified by group affiliation.

Possible confounding was assessed by robust regression and considered relevant if the outcome of the model was changed by at least 25% [26] by any of the possible confounders (socioeconomic factors, parental allergic disease, secondary smoke). Confounding by socioeconomic factors was tested by including these factors in multiple regression models, and eliminating the factors with highest p-values stepwise by Hosmer’s procedure [26] until only factors with p-values < 0.05 remained, retaining age and gender.

The level of statistical significance was set to p<0.05 for all analyses.

Analyses were performed with the IBM SPSS Statistics 21 (IBM Corporation, Armonk, New York, USA), and the NumberCruncher Statistical System (NCSS Kaysville, Utah, USA), version 11.

## Results

Children in the bronchiolitis group were significantly more often affected by at least one atopic manifestation at two years of age and had more often rBO than the controls, while AD was similar in the two groups (Table 1).

### Quality of life, salivary cortisol and atopic manifestations at two years of age

The QoL_2_ scores varied from 0–100 in five domains, with the smallest score range seen in the domain Getting along (53.3), as shown in Table 2. The bronchiolitis group had a larger reported improvement in health, Change in health, compared with one year ago, while controls scored significantly higher for Overall health and General health (Table 2).

**Table 2:** Unadjusted weighted means (95% CI) of QoL at two years of age (QoL24m) of children included at hospitalisation for acute bronchiolitis and control children, and descriptive QoL data of all children.

Domain	Previous bronchiolitis	Control children	All children
	Unadjusted weighted means (95% CI)		Median (min, max)
Overall health	83.4 (81.3, 85.5)**	88.7 (86.3, 91.1)	85.0 (0.0, 100.0)
Physical abilities	100.0 (100.0, 100.0)	100.0 (100.0, 100.0)	100.0 (0.0, 100.0)
Growth and development	94.7 (93.7, 95.6)	95.2 (94.1, 96.3)	97.2 (0.0, 100.0)
Bodily pain/ discomfort	80.5 (78.4, 82.6)	78.5 (76.0, 80.9)	75.0 (8.3, 100.0)
Temperament and moods	84.2 (83.0, 85.4)	83.0 (81.7, 84.4)	84.7 (36.8, 100.0)
General behaviour	84.5 (82.9, 86.0)	85.2 (83.4, 86.9)	85.4 (35.4, 100.0)
Overall behaviour	85.0 (85.0, 85.0)	85.0 (85.0, 85.0)	85.0 (30.0, 100.0)
Getting along	78.8 (77.6, 80.0)	78.5 (77.2, 79.9)	78.3 (45.0, 98.2)
General health	67.1 (65.0, 69.2)****	78.3 (75.9, 80.7)	75.0 (18.2, 100.0)
Change in health	65.2 (62.8, 67.8)****	56.9 (54.1, 59.7)	50.0 (0.0, 100.0)
Parental impact—emotions	91.3 (90.1, 92.6)	91.3 (89.9, 92.7)	92.9 (35.7, 100.0)
Parental impact—time	95.2 (94.2, 96.1)	94.2 (93.1, 95.3)	95.2 (28.6, 100.0)
Family cohesion	79.6 (77.3, 82.0)	80.8 (78.1, 83.5)	85.0 (0.0, 100.0)

** p<0.01

****p<0.0001

**Fig 3:**
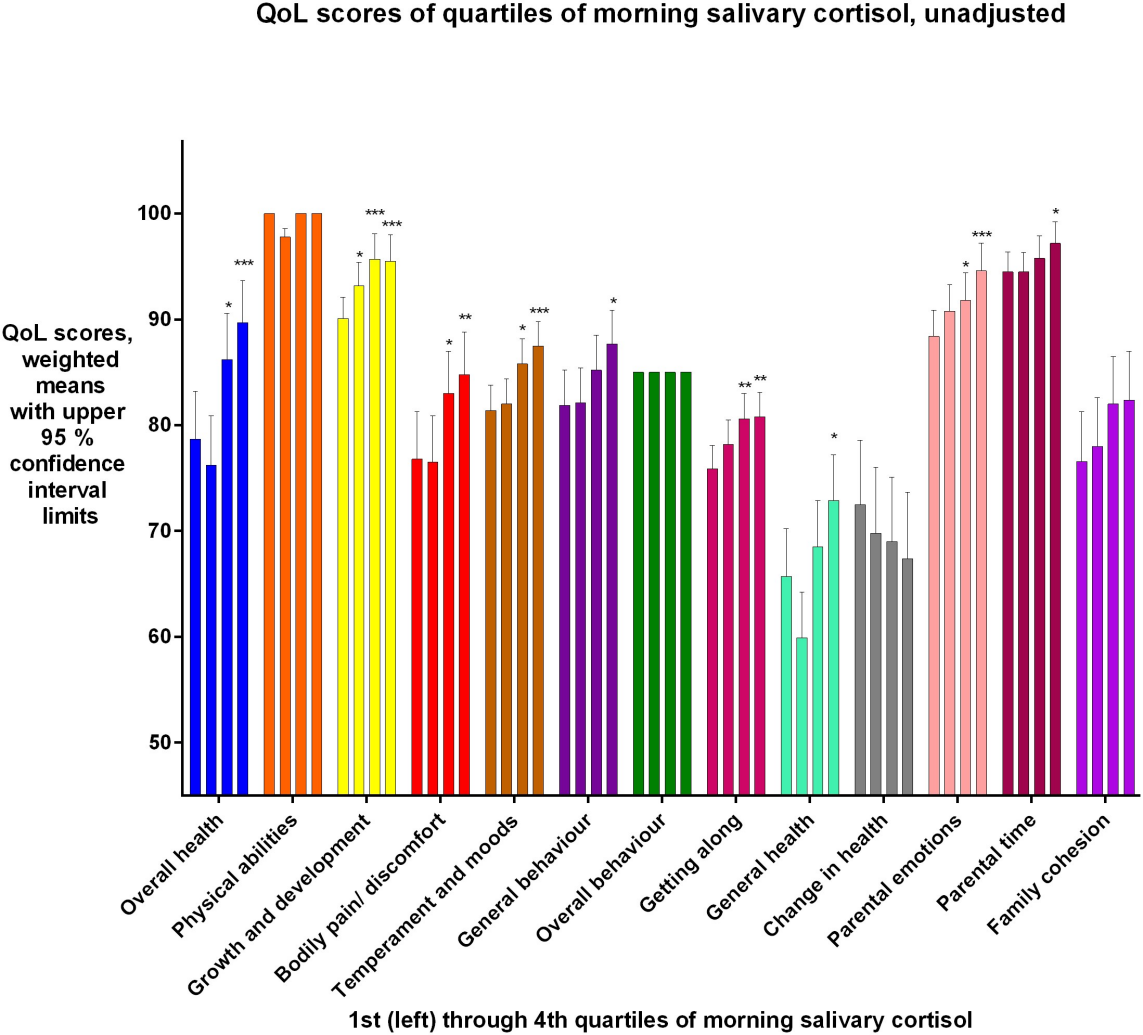
Bronchiolitis group: QoL_2_ scores for each domain, unadjusted, for each quartile of morning salivary cortisol, 1^st^ quartile lowest cortisol, 4^th^ quartile highest. Due to interaction between gender and cortisol for the Overall health domain, this domain was analysed separately for the genders. An association was found only for boys for this domain. For Overall health, results for boys are shown. For the other domains, results for both genders analysed together are shown.

Eight QoL_2_ domains were significantly reduced with decreasing salivary cortisol levels (p = 0.0001-p = 0.035) among the bronchiolitis group, see Table 3 and Fig 3 In the same group, the association between Overall health and salivary cortisol was significant in boys only, (p<0.0001).

**Table 3:** The potential influence of recurrent bronchial obstruction (rBO), atopic dermatitis (AD) and allergic sensitisation (AS) on the associations between Quality of Life (QoL_2_) and salivary cortisol at two years of age is shown for 203 children who had moderate to severe acute bronchiolitis in infancy. The influence by including each atopic manifestation (rBO, AD and AS) is shown as the percentage change of QoL per 1 nmol/L change in salivary cortisol, adjusted for age and gender. Each column includes all children with the observed atopic manifestation, and they are not mutually exclusive.

Domain (Mean domain score difference^1^ by difference between 95^th^ and 5^th^ percentile of cortisol, 51.6 nmol/L)	Change in QoL_2_ score per nmol/L unit salivary cortisol	rBO	AD	Allergic sensitisation
		% change in association		
Overall health^2^ boys (16.0)	0.31 (0.17, 0.45)****	-20.7	-2.1	-0.5
Overall health girls (-0.0)	-0.00 (-0.16, 0.16)			
Growth and development (3.8)	0.07 (0.02, 0.13)**	-1.4	1.7	0.6
Bodily pain/ discomfort (6.2)	0.12 (0.01, 0.23)*	-8.3	5.4	-2.3
Temperament and moods (6.1)	0.12 (0.06, 0.18)***	-6.9	1.4	-1.5
General behaviour (4.6)	0.09 (0.01, 0.17)*	-6.7	-1.5	1.3
Getting along (4.0)	0.08 (0.02, 0.13)**	-3.0	-0.3	0.9
General health (5.6)	0.11 (-0.00, 0.22)^3^	-26.9	0.4	0.3
Parental impact—Emotions (4.5)	0.09 (0.3, 015)**	-6.1	-5.1	1.2
Parental impact—Time (3.0)	0.06 (0.01, 0.10)*	-7.7	-0.1	0.9

^1^QoL score difference equals percentage point difference.

^2^Overall health was gender stratified due to interaction.

*p<0.05

**p<0.01

*** p<0.001

****p<0.0001

^3^p = 0.0517

In the controls, General health only was significantly associated and was lower with lower cortisol. The significant decrease of 0.1 percentage point per nmol/L in cortisol level (95% CI 0.0, 0.2, p = 0.046) corresponded to a QoL_2_ difference of five percent points between children having cortisol levels at the 5^th^ vs 95^th^ percentile (a difference of 51.6 nmol/L of salivary cortisol). No further analyses were performed in this group, with only one QoL domain significantly associated with salivary cortisol.

The hypothesised (top) and observed (bottom) influence of atopic manifestations on cortisol and QoL_2_ are shown schematically in Fig 2. The strongest influence on the associations between cortisol and QoL_2_ was exerted by rBO, reducing the associations with 1.4 to 26.9 per cent, followed by changes related to AD ranging from -5.5 to 5.1 and less than 3 per cent changes by allergic sensitisation (Table 2), with all associations between QoL and cortisol remaining significant after including rBO, AD and allergic sensitisation into the regression analyses (results not shown). The To illustrate the combined estimated effect from rBO and cortisol levels on the Overall health domain, a 24-months-old boy with rBO and low salivary cortisol, at the 5^th^ percentile, would have an estimated 23.1 percentage point lower QoL than a boy without rBO who had a high salivary cortisol level, at the 95^th^ percentile.

We found no significant confounding effect of socioeconomic factors, parental ethnicity and second-hand smoke at two years of age, and these were consequently not included in the final multivariate analyses (Table 4).

**Table 4:** Bronchiolitis group: Change of associations between salivary morning cortisol at and QoL_2_ at two years of age by socioeconomic factors, including age, gender, and the following socioeconomic factors: Mother’s education, father’s education, mother’s income, father’s income, ethnicity of father and of mother (Caucasian or not) and secondhand smoke exposure at two years of age. The socioeconomic factors have been eliminated by Hosmer’s stepdown procedure, finally retaining factors with p<0.05. Age and gender have been retained in the models.

Adjusted for/domain	Change of QoL score per nmol/L changed salivary cortisol after adjustment	% influence on change of QoL score by adjustment	Socioeconomic factors retained in the model
Overall Health	0.15 (0.05, 0.26)**	-16.7%	Caucasian father^1^ ****
Growth and Development	0.07 (0.02, 0.13)*	-3.7%	Caucasian father^1^*
Bodily Pain/ Discomfort	0.12 (0.01, 0.23)*		All factors insignificant; eliminated from model
Temperament and Moods	0.11 (0.05, 0.17)***	-8.1%	Caucasian mother^1^**
General Behaviour	0.08 (0.00, 0.16)*	-10.9%	Caucasian mother^1^**
Getting Along	0.06 (0.01, 0.12)*	-18.8%	Education mother^1^**, education father^2^**, Caucasian mother^1^**
Parental Impact—Emotions	0.08 (0.02, 0.14)*	-11.7%	Income father^1^*, Caucasian mother^1^****
Parental Impact—Time	0.05 (0.01, 0.10)*	-13.1%	Caucasian mother^1^* Caucasian father^1^**

^1^ positively associated with QoL domain

^2^ negatively associated with QoL domain

*p<0.05

**p<0.01

***p<0.001

****p<0.0001

The age and gender adjusted salivary cortisol levels at two years were similar in the bronchiolitis group and controls, with a non-significant weighted mean difference of -0.70 (95% CI -3.7, 2.3) nmol/L.

**Fig 4:**
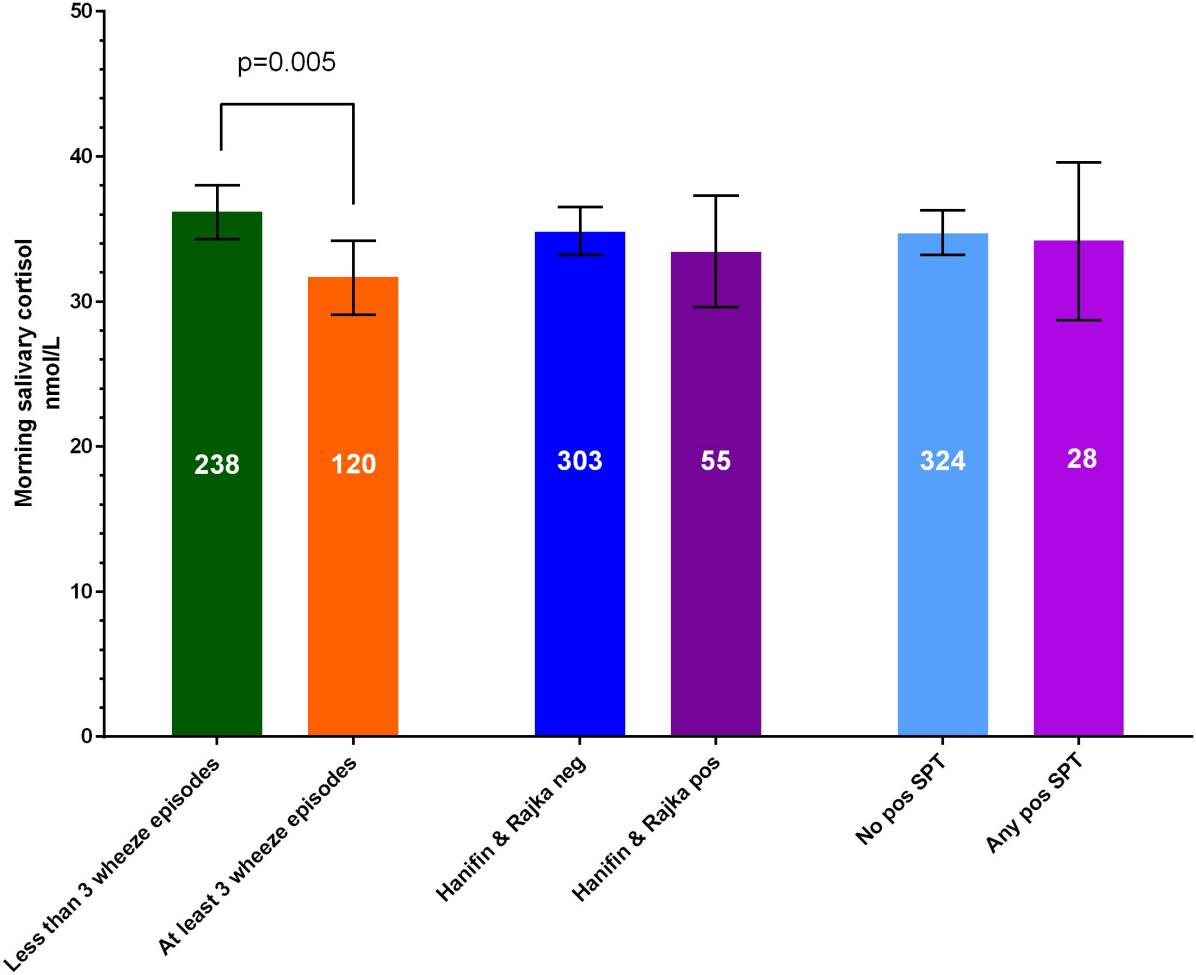
Morning salivary cortisol in children of the bronchiolitis group and controls together, without recurrent bronchial obstruction (rBO), defined as at least three wheeze episodes, compared to with rBO, no AD vs. with AD as well as with no or any positive skin prick test (SPT) to common inhalant and food allergens.

Salivary cortisol was significantly lower among the 120 children with rBO compared to the 238 children without rBO (weighted mean difference -4.1 (95%CI -7.3,-1.0) nmol/L), as shown schematically in Fig 2, and in unadjusted analysis in Fig 4. Neither AD nor allergic sensitisation was significantly associated with morning salivary cortisol at two years of age (Figs 2 and 4).

The QoL_2_ was significantly associated with rBO and AD in the bronchiolitis group and with rBO as well as allergic sensitisation in the controls, as reported in Table 5 by percentage point changes in QoL_2_ scores by the atopic diseases.

**Table 5:** The impact of allergic diseases on QoL_2_ is given for each domain as mean difference from not having the condition, given as percentage points (95% CI), adjusted for age and gender. Results are stratified for enrolment group based upon interaction analyses. As an example; the negative association of General health (GH) with rBO is stronger in the bronchiolitis group than among controls, both being statistically significant.

	Recurrent bronchial obstruction		Atopic dermatitis		Allergic sensitisation	
	bronchiolitis group	controls	bronchiolitis group	controls	bronchiolitis group	controls
**OH**	-12.3 (-16.5, -8.2)****	-6.6 (-13.9, 0.7)	-5.5 (-11.7, 0.6)	-6.3 (-13.6, 0.9)	-0.7 (-8.6, 7.2)	-11.7 (-21.6, -1.9)*
**PA**	-1.2 (-2.1, -0.4)**	Not done	-0.0 (-0.0, 0.0)	-2.1 (-3.2, -1.0)***	0.0 (-0.2, 0.2)	Not done
**GD**	-3.5 (-5.8, -1.2)**	-1.3 (-4.3, 1.8)	-2.5 (-5.8, 0.7)	-1.2 (-4.2, 1.8)	-0.1 (-4.3, 4.0)	-1.9 (-6.4, 2.6)
**BD**	-6.5 (-10‥8, -2.1)**	-7.3 (-14.7, 0.1)	-7.2 (-13.4, -0.9)*	-1.7 (-8.6, 5.3)	4.2 (-3.6, 12.1)	-6.4 (-16.8, 4.0)
**TM**	-3.1 (-5.5, -0.7)*	-4.1 (-7.8, -0.5)*	-5.1 (-8.4, -1.8)**	1.0 (-2.6, 4.5)	-2.5 (-1.8, 6.8)	-1.3 (-6.5, 3.9)
**GB**	-4.7 (-7.9, -1.4)**	-0.7 (-5.5, 4.1)	-5.3 (-9.9, -0.7)*	-4.1 (-8.8, 0.6)	-0.8 (-6.8, 5.2)	-7.0 (-13.7, -0.3)*
**OB**	-0.0 (-0.0, 0.0)	Not done	0.0 (0.0, 0.0)	Not done	0.0 (-0.0, 0.0)	Not done
**GA**	-1.9 (-4.2, 0.4)	-4.0 (-7.9, -0.0)*	-6.2 (-9.3, -3.0)***	-0.9 (-4.8, 2.9)	-0.4 (-4.6, 3.8)	-8.0 (-13.6, -2.4)**
**GH**	-13.8 (-17.8, -9.8)****	-6.8 (-12.9–0.7)*	-5.8 (-12.0, 0.3)	-7.0 (-13.0, -1.0)*	0.1 (-7.9, 8.1)	-12.2 (-20.5, -3.9)**
**CH**	6.9 (0.8, 13.0)*	6.8 (-0.6, 14.2)	7.8 (-0.9, 16.4)	3.1 (-3.3, 9.6)	-1.0 (-12.3, 10.3)	7.3 (-2.8, 17.4)
**PE**	-4.0 (-6.4, -1.5)**	-2.3 (-5.9, 1.4)	-5.7 (-9.2, -2.2)**	-1.6 (-5.2, 2.0)	-0.2 (-4.7, 4.3)	-5.8 (-10.7, -1.0)*
**PT**	-2.5 (-4.5, -0.5)*	-2.1 (-5.0, 1.0)	-1.9 (-4.8, 1.0)	-1.9 (-4.9, 1.0)	-0.0 (-3.7, 3.7)	-4.3 (-8.3, -0.2)*
**FC**	-0.1 (-4.7, 4.5)	0.3 (-7.4, 8.0)	-5.4 (-11.9, 1.1)	0.7 (-6.7, 8.1)	-0.0 (-3.7, 3.7)	-1.8 (-12.3, 8,7)

Not done refers to analyses that were not applicable due to strong correlations between some of the included variables.

*p<0.05

**p<0.01

***p<0.001

p<0.0001

OH = Overall health; PA = Physical abilities; GD = Growth and development; BD = Bodily pain/ discomfort; TM = Temperament and moods; GB = General behaviour; OB = Overall behaviour; GA = Getting along; GH = General health; CH = Change in health; PE = Parental impact—emotions; PT = Parental impact—time; FC = Family cohesion

### QoL_1_ and salivary cortisol at two years

Lower morning salivary cortisol at two years of age was significantly associated with lower QoL nine months after enrolment within the bronchiolitis group by age and gender adjustment, as shown in Table 6. No significant associations were observed between morning salivary cortisol and QoL_1_ among the controls.

**Table 6:** Significant associations between QoL_1_ (at a mean age of 14 months and morning salivary cortisol at two years are presented as the mean age and gender adjusted change in cortisol level per change scores per domain.

	Change in cortisol nmol/L per QoL14m score change
Overall health	0.17 (0.02, 0.32)*
Physical abilities	0.92 (0.41, 1.43)***
Growth and development	0.34 (0.09, 0.60)**
Temperament and moods	0.35 (0.12, 0.59)**
General health	0.17 (0.01, 0.33)*
Parental impact—emotions	0.45 (0.19, 0.71)***
Parental impact—time	0.28 (0.11, 0.46)**

*p<0.05

**p<0.01

***p<0.001

## Discussion

By this explorative add-on to the Bronchiolitis All SE-Norway study, including children enrolled at hospitalisation for acute bronchiolitis and control children, we confirmed the hypothesis that *low* cortisol levels at visits not requiring acute hospitalisation may contribute to development of asthma in children with moderate to severe acute bronchiolitis in infancy, but not in controls. The observed reduction in most QoL domains with lower cortisol levels was partly explained by rBO, whereas the impact of atopic dermatitis was less clear. No influence was observed by allergic sensitisation. Furthermore, we could confirm our second hypothesis, that reduced QoL some months after hospitalisation for moderate to severe bronchiolitis may be a marker of chronic stress, by demonstrating that lower QoL at a mean age of 14 months was associated with lower cortisol levels at two years of age. Finally, the associations at two years of age between cortisol and QoL could only partly be explained by rBO.

We are not aware of other studies comparing QoL and morning salivary cortisol in children. We have previously found that infants with acute bronchiolitis have higher morning salivary cortisol than controls [23], indicating acute stress. Others have found other signs of acute stress in acute bronchiolitis with respiratory syncytial virus, differing from other infections and acute diseases [27]. An explanation of a seeming contradiction, that at inclusion with acute bronchiolitis the cortisol levels were higher, but later lower in subjects with low QoL as signs of chronic stress, can be that these infants, while hospitalised with moderate to severe bronchiolitis, had not yet developed asthma or were not hit by a blunted cortisol response seen in asthmatic children with time, or were so profoundly affected by acute stress that even subjects with a blunted cortisol response managed to raise cortisol. Reduced basal morning cortisol levels observed in children with asthma, also without concurrent use of inhaled corticosteroids (ICS) [3], may on the other hand indicate chronic immunological stress, see Supporting Information. The subsequent blunted cortisol responses to acute stress reported in subjects with asthma related to a disturbance of the hypothalamus-pituitary-adrenal (HPA) axis differs from the chronic stress in non-atopic children that can lead to a higher cortisol response [20, 28]. A possible connection between our finding of lower morning salivary cortisol in children with lower QoL in the bronchiolitis group whereas others found lower cortisol responses to acute stress in children with asthma [20] can be that asthmatic children may not only have a blunted response to acute stress, but also a lower diurnal variation as well as a possibly lower cortisol awakening response, which is influenced by acute stress [29]. In line with our findings, a tendency for decreased cortisol levels has been found in young children with at least three wheeze episodes [30]. A possible explanation why a lower cortisol response to acute stress in children and adults with AD was found by Kojima et al. [31], but not in our nor another study, can be that AD had lasted longer or were more serious than in our or the ALADDIN cohort study [30].

Reduced QoL after acute bronchiolitis [13, 32], may partly be expressions of chronic physical, and psychological stress. Concerns of the parents of the children of the bronchiolitis group, as indicated by the Parental impact—emotions and Parental impact—time domains in the present study, seem to be associated with the children’s cortisol levels. The associations between cortisol and QoL in domains reflecting expressions of pain, moods and behaviour, i.e. Bodily pain/ discomfort, Temperament and moods, General behaviour and Getting along, partly influenced by rBO, may also indicate a role of psychological stress in the development of asthma.

The 16 percentage point difference in Overall health in boys with low versus high salivary cortisol is likely to be clinically relevant as they are comparable to the eight percentage point General health differences between children with and without asthma-like symptoms reported from the Generation R study [33].

The observed association between Overall health and morning salivary cortisol at two years of age was significant among both genders analysed together, but only in boys by gender stratified analyses performed for this domain due to interaction. This may be related to our finding of significantly higher salivary cortisol levels in girls compared to boys at two years in the Bronchiolitis all SE-Norway study [23].

The lack of significant associations between allergic sensitisation and QoL in the bronchiolitis group and between allergic sensitisation and salivary cortisol may have several explanations. In our study less than 10 per cent of the subjects were sensitised to at least one allergen, limiting the likelihood of observing significant associations. On the other hand, allergic sensitisation may not affect QoL before allergen exposure causes symptoms, which for inhalant allergens occur more frequently with increasing age [34].

Our finding that reduced QoL about one year of age was associated with lower salivary cortisol at two years of age is consistent with our recently published finding in the same study population that in addition to having been hospitalised for acute bronchiolitis, disease severity and asthma risk factors as well as AD at inclusion were associated with reduced QoL at 14 months of age [8, 21].

The direct clinical implications of our findings remain unclear at present. The maintenance of statistical significance of the influence of cortisol on QoL after adjusting for rBO indicates a role of other factors than an obvious proxy for asthma on the concordant relationship between morning salivary cortisol and QoL after acute bronchiolitis. However, our study suggests that in addition to rBO, also acute moderate to severe infant bronchiolitis, disposing for asthma, may play a role in the association between future salivary cortisol and QoL in subjects who have not yet developed asthma. Although the influence of the asthma proxy rBO dominated the association between cortisol and QoL at two years, the associations were significant also among children in the bronchiolitis group without rBO, see Supporting Information. Together these observations suggest that children who have acute bronchiolitis in infancy and reduced QoL some months later may be vulnerable to chronic stress, observed by lower salivary cortisol and reduced QoL at two years of age. Thus, our study supports a role of chronic stress indicated by lower cortisol levels in development of asthma.

In line with previous studies finding marginally lower cortisol in adolescents with low socioeconomic status [35], we included socioeconomic data as well as second-hand smoking into regression analyses. However, none of these factors were found to be significant confounders, possibly reflecting the overriding effects by atopic diseases in the children, as well as a low frequency of second-hand smoke in our cohort.

### Strengths and limitations

The study strengths include a prospective design of a reasonably large group of children included in infancy with acute bronchiolitis and atopic disease, a control group of similar age recruited from a general population with a frequency of atopic manifestations (rBO, AD and allergic sensitisation) on the same levels as other two year old children in Norway [36], a high retention rate at follow-up investigations, repeated measurements and stringent clinical characterisation of the subjects. Also, the findings appear robust, as the associations remained significant after relevant adjustments.

The lack of significant associations between QoL and salivary cortisol in the control group may be due to the relatively few subjects with rBO, most consistently associated with reduced QoL and salivary cortisol, and that the control children may be more heterogeneous, possibly with a lower risk of future asthma development, or that the control children in general had a higher QoL.

As previously reported [8, 21], we decided a priori not to adjust for multiple analyses, as the QoL domains were not independent from each other. Also, the associations with the different QoL domains point in the same direction, limiting the likelihood of incidental findings. As expected, the Change in health domain improved more among children with previous acute bronchiolitis than among controls, in line with findings in children with chronic diseases [13].

Our use of single morning salivary cortisol measurements may be a limitation in terms of identifying diurnal variation, but improved feasibility of obtaining such measurements. However, previous studies of single morning measurements [3] and the lack of significant day-to-day variation between three samples taken at 4- to 8-day intervals [37], suggest that single measures may reflect habitual morning cortisol. We sampled as soon as possible after the first awakening after 6:00 a.m. [23], possibly encompassing a morning awakening response and the top circadian morning cortisol [38].

### Conclusion

At two years of age most QoL domainsdecreased with lower morning salivary cortisol among children who had been hospitalised for acute bronchiolitis in infancy, but for one domain only among controls. Recurrent bronchial obstruction and, to a limited extent atopic dermatitis weakened these associations that nevertheless remained significant. After bronchiolitis, lower QoL in one-year-old children was associated with lower salivary cortisol at two years.

## Supporting information

S1 Table Severity of the allergic diseases of the 358 children of the study population. (DOCX)

S2 Table Per cent change of the association between QoL24m and morning salivary cortisol at two years of age, by adjusting for the total number of wheeze episodes, SCORAD index and the sum in mm of positive skin prick tests (except for histamine) in addition to age and gender, bronchiolitis group. Only domains with statistically significant associations are presented.
(DOCX)

S3 Table Associations between QoL24m and cortisol, nmol/L (95% CI), adjusted for age and gender in children without RBO, bronchiolitis group; only domains significantly associated with cortisol when children with RBO are included are shown. (DOCX)

S1 File (DOCX)

## Abbreviations

AD: Atopic dermatitis
ITQOL: The Infant Toddler Quality of Life Questionnaire
QoL: Health related quality of life
