# Supplementary material for: Quality of life, salivary cortisol and atopic diseases in young children
Source: PLoS One. 2019 Aug 30;14(8):e0214040. doi: 10.1371/journal.pone.0214040 (PMC6716779; doi:10.1371/journal.pone.0214040)
Supplement: S1 Table — (DOCX) [file pone.0214040.s001.docx]

**S1 Table**

**Severity of the allergic diseases of the 358 children of the study population**

|  | Bronchiolitis group^1^  N=206 | Control group  N=152 |
| --- | --- | --- |
| SCORAD^1^, mean, SD | 7.0 (9.5) | 7.8 (13.7) |
| SCORAD 0 n (%) | 102 (49.5%) | 97 (63.8%) |
| Mild AD (SCORAD<25) n (%) | 91 (44.2%)** | 40 (26.3%) |
| Moderate AD (SCORAD 25-50) n (%) | 12 (5.8%) | 10 (6.6%) |
| Severe AD (SCORAD>50) n (%) | 1 (0.5%) | 5 (3.3%) |
| Total no. of wheeze episodes, mean (SD) | 5.7 (8.4)*** | 1.1 (2.1) |
| Sum SPT^2^, mean, SD | 1.3 (3.3) | 1.5 (4.2) |

^1^SCORAD rating took place when signs of AD were seen. No SCORAD data were set to zero.

^2^Sum of wheal diameters, exceeding negative controls, histamine controls not included


* p<0.05 ** p<0.01 ***<0.001
