# Supplementary material for: Quality of life, salivary cortisol and atopic diseases in young children
Source: PLoS One. 2019 Aug 30;14(8):e0214040. doi: 10.1371/journal.pone.0214040 (PMC6716779; doi:10.1371/journal.pone.0214040)
Supplement: S2 Table — Only domains with statistically significant associations are presented. (DOCX) [file pone.0214040.s002.docx]

**S2 Table**

**Per cent change of the association between QoL24m and morning salivary cortisol at two years of age, by adjusting for the total number of wheeze episodes, SCORAD index and the sum in mm of positive skin prick tests (except for histamine) in addition to age and gender, bronchiolitis group. Only domains with statistically significant associations are presented.**

|  | Change by adjustment for no. of wheeze episodes | Change by adjustment for SCORAD | Change by adjustment for the sum of positive SPT wheal diameters | Change by adjustment for no. of wheeze episodes, SCORAD and the sum of positive SPT wheal diameters |
| --- | --- | --- | --- | --- |
| Overall health | -22.8 % | -4.6 % | 0.5 % | -25.1 % |
| Growth and development | -16.6 % | 1.2 % | 0.4% | -13.0 % |
| Bodily pain/ discomfort | -25.9 %^1^ | 3.1 % | -1.1 % | -21.4 %^1^ |
| Temperament and moods | -15.7 % | 0.8 % | 0.0 % | -12.9 % |
| General behaviour | -13.2 %^1^ | -0.5 % | -0.6 % | -13.2 %^1^ |
| Getting along | -8.1 % | 3.4 % | 0.4 % | -7.3 % |
| Parental impact - emotions | -17.5 % | 3.1 % | -1.1 % | -14.7 % |
| Parental impact - time | -16.7 % | -0.5 % | 1.3 % | -11.9 % |

^1^No longer statistically significant after adjustment

.
