# Supplementary material for: Quality of life, salivary cortisol and atopic diseases in young children
Source: PLoS One. 2019 Aug 30;14(8):e0214040. doi: 10.1371/journal.pone.0214040 (PMC6716779; doi:10.1371/journal.pone.0214040)
Supplement: S3 Table — (DOCX) [file pone.0214040.s003.docx]

**S3 Table**

**Associations between QoL24m and cortisol, nmol/L (95% CI), adjusted for age and gender in children without RBO, bronchiolitis group; only domains significantly associated with cortisol when children with RBO are included are shown.**

| Domain | n | Change QoL score per nmol/L cortisol |
| --- | --- | --- |
| Overall health | 95 | 0.14 (-0.01, 0.28) |
| Overall health boys^1^ | 50 | 0.31 (0.13, 0.50)** |
| Overall health girls | 45 | 0.01 (-0.21, 0.23) |
| Growth and development | 103 | 0.07 (0.01, 0.14)* |
| Bodily pain/ development | 104 | 0.14 (-0.01, 0.28) |
| Temperament and moods | 105 | 0.10 (0.02, 0.18)* |
| General behaviour | 105 | 0.10 (-0.00, 0.21) |
| Getting along | 103 | 0.06 (-0.02, 0.14) |
| Parental impact – emotions | 104 | 0.09 (0.01, 0.16)* |
| Parental time | 102 | 0.07 (0.01, 0.12)* |

^1^Stratified for genders due to interaction

* p<0.05 ** p<0.01
