# Supplementary material for: Quality of life, salivary cortisol and atopic diseases in young children
Source: PLoS One. 2019 Aug 30;14(8):e0214040. doi: 10.1371/journal.pone.0214040 (PMC6716779; doi:10.1371/journal.pone.0214040)
Supplement: S1 File — (DOCX) [file pone.0214040.s004.docx]

# **Quality of life, salivary cortisol and allergic diseases in young children Supporting Information**

## **Study design**

### Inclusion criteria

Inclusion criteria for the bronchiolitis group were clinical signs of bronchiolitis, as defined by Court: Illness mainly affecting infants, especially in the first 6 months of life. Rapid respiration, dyspnea, wheezing, chest recession, cough, rhonchi and rales are very frequent. Visible distension of the chest and increased pulmonary translucency on the chest radiograph are frequent and of high diagnostic significance. Upper respiratory features, especially nasal discharge and a red pharynx are frequent. Fever is very frequent, but high fever uncommon ([1](#_ENREF_1)). To be enrolled at hospital admission for acute bronchiolitis, the bronchiolitis had to be rated as moderate to severe, with at least 4 of a maximum of 10 points of a scale published by S. Kristjansson et al. ([2](#_ENREF_2)), and described by H.O. Skjerven et al. ([3](#_ENREF_3)), and the age should be below 12 months of age. Inclusion criteria for the children of the general population ([4](#_ENREF_4)) was age below 12 months.

### Exclusion criteria

For the bronchiolitis group, exclusion criteria were any severe or chronic disease that might significantly influence the progression of acute bronchiolitis, more than one previous episode of obstructive airways disease or more than four weeks with lower airways disease symptoms and, or, use of inhaled corticosteroids in the previous four weeks. Exclusion criteria for the population based control group were any severe underlying disease, for example heart, lung, immunological, neurological or oncological disease.

## **Subjects**

Significantly more of the subjects from the control group met at the two year of age follow-up examination and yielded QoL answers as well as successful cortisol samples, than of the bronchiolitis group. From the control group, we obtained QoL and cortisol results from 155 children, 64.6 %, and from the bronchiolitis group, from 203 children, 50.2 %. Children without QoL or salivary cortisol data or who did not meet at two years of age (201 from the original bronchiolitis group and 85 controls) differed from those who met when it came to parental education (mothers 3.87 vs. 4.34, p<0.001, fathers 3.87 vs. 4.08, p<0.01), ethnicity (Caucasian mother 89.7 % vs. 94.4 %, p<0.05, Caucasian father (88.8 % vs. 94.4 %, p <0.01), and smoking in the homes 15,3 % vs. 9.2 %), but not with respect to parental asthma, parental rhinoconjunctivitis or atopic dermatitis at enrolment.

## **Methods**

### Recurrent bronchial obstruction (rBO)

At the follow-up visit, the parents were asked about the number of wheezing episodes in their child’s first year and second year, to provide a measure of asthma severity for supplementary analyses. At enrolment, the parents had been asked if their child had had more than one episode of wheezing earlier, because this was an exclusion criterion for the bronchiolitis group. The parents of the control subjects were asked about the number of wheeze episodes also at enrolment in infancy. The number of wheezing episodes was adjusted for information at enrolment, in case this was forgotten, and an episode of bronchiolitis was counted as one episode of wheezing.

### Hanifin and Rajka criteria for AD

We defined fulfilment of the criteria this way: 1. At least three of the four main criteria: pruritus; dermatitis in the face and/or the extensor surfaces of the extremities; chronic or relapsing dermatitis; personal or family history of atopy (asthma, allergic rhinitis, AD). 2. In addition, at least three minor features: Onset in childhood; deterioration by emotional factors; deterioration by environmental factors; products may irritate the skin, e.g. wool, soap; tendency toward cutaneous infections with virus and bacteria; facial pallor or facial erythema; hypopigmented spots; dark skin below the palpebral fissures; Morgan infraorbital fold; angular cheilitis; anterior neck folds; ichthyosis; palmar hyperlinearity; keratosis pilaris; dermatitis on hands and feet (“winter feet”); white dermographism; perifollicular accentuation; nipple eczema; recurrent conjunctivitis; dry skin.

Compared to the original Hanifin and Rajka criteria; five of the original 23 minor features were not asked or examined for: The two ophthalmological criteria originally included by Hanifin and Rajka, keratoconus and anterior or subcapsular cataracts, were not included and would not have been likely to have given any positive answers. Some of the side criteria were grouped together as one criterion in the original definition. Furthermore, food intolerance, elevated serum IgE or type I skin reactivity were not included, but type I skin reactivity was included as an own atopic manifestation not included in the definition, as described in the main text.

Severity assessment of atopic dermatitis

Severity was rated by the SCORing AD (SCORAD) index ([5](#_ENREF_5)), in all children with signs of AD. The higher of the two values, obtained by a trained physician and a trained nurse was used for analyses. In unrated children, the indexes were set to zero.

Allergic sensitisation

Allergic sensitisation was determined by skin prick test (SPT) was tested with the following allergens: dog dander, cat dander, house dust mite (Dermatophagoides pteronyssinus), birch, timothy and mugwort pollen, the mould species Cladosporium herbarum and Alternaria tenuis, hen’s egg white, peanut, almond, hazelnut, wheat, cow’s milk, soy, cod, shrimp and positive and negative controls (Soluprick, Soluprick SQ, ALK, Hørsholm, Denmark). The sum of allergen wheal diameters exceeding negative control ([6](#_ENREF_6)) were included in disease severity analyses,

## **Results**

Median (min.-max.) duration from this first QoL (QoL_1_) survey to the follow-up clinical visit and second QoL survey (QoL_2_) with the same questionnaire was 9.6 (5.4-13.8) months.

RBO was negatively associated with nine QoL_2_ domains of the bronchiolitis group and three domains of the control group, and positively with one domain of the bronchiolitis group, Change in health (compared to one year ago), AD negatively with QoL_2_ in seven domains in the bronchiolitis group and in one domain in the control children, whereas allergic sensitisation was not significantly associated with any QoL_2_ domain in the bronchiolitis group, but negatively with six domains in the control group.

S1 Table indicates the severity of the allergic diseases and S2 Table show results from adjusting for the continuous expressions of the atopic manifestations. In the bronchiolitis group, adjusting for the number of wheeze episodes, reduced the associations between cortisol and QoL further for seven domains. Statistical significance disappears for Bodily pain/ discomfort and General behaviour, illustrating the impact by wheeze on associations between these QoL_2_ domains and cortisol. Adjustment for the severity of AD or degree of allergic sensitisation did not turn any associations to insignificance. Also in children without rBO, we found an association in the bronchiolitis group between cortisol and QoL_2_ in the domains Overall health (boys), Growth and development, Temperament and moods, General behaviour, Parental impact – emotions and Parental impact – time. Details are shown in S3 Table.

## **Immunological aspects**

The severity of acute bronchiolitis has been associated with plasma cortisol suppressing the T-helper cell type 1 (Th1) immune response ([7](#_ENREF_7)), possibly leading to a shift to a Th2 response in acute bronchiolitis through inhibition of the interferon gamma response acting directly on T cells or indirectly through IL-12. Glucocorticoids may stimulate the secretion of IL-4 and IL-10, enhancing the Th-2 response, and stimulate Th2-cells directly ([8](#_ENREF_8)), as well as possibly supressing Th2-inflammation ([9](#_ENREF_9)).

## **The Bronchiolitis Study Group**

Håvard Ove Skjerven^9,1^, Jon Olav Gjengstø Hunderi^2^, Sabine Kristin Brügmann-Pieper^3^, Anne Charlotte Brun^4^, Kai-Håkon Carlsen^1,9^, Hanne Engen^5^, Leif Eskedal^6^, Marius Haavaldsen^2^, Bente Kvenshagen^2^, Jon Lunde^2^, Petter Mowinckel^1^, Leif Bjarte Rolfsjord^7^, Christian Siva^4^, Truls Vikin^8^, Karin C. Lødrup Carlsen^1,9^.

1. Department of Paediatrics, Oslo University Hospital, 2) Department of Paediatrics , Østfold Hospital HF, 3) Department of Paediatrics , Vestre Viken HF Drammen, 4) Department of Paediatrics , Vestfold Hospital HF Tønsberg, 5) Department of Paediatrics , Telemark Hospital Skien, 6) Department of Paediatrics , Sørlandet Hospital HF, Kristiansand, 7) Department of Paediatrics , Innlandet Hospital HF Elverum, 8) Department of Paediatrics, Innlandet Hospital HF Lillehammer, 9) Faculty of Medicine, University of Oslo.

Study nurses at Oslo University Hospital

Solveig Knutsen, Live S. Nordhagen and Liv Julie Sørdal

## **References**

1. Court SD. The definition of acute respiratory illnesses in children. Postgrad Med J. 1973;49(577):771-6.

2. Kristjansson S, Lodrup Carlsen KC, Wennergren G, Strannegard IL, Carlsen KH. Nebulised racemic adrenaline in the treatment of acute bronchiolitis in infants and toddlers. Archives of disease in childhood. 1993;69(6):650-4.

3. Skjerven HO, Hunderi JO, Brugmann-Pieper SK, Brun AC, Engen H, Eskedal L, et al. Racemic adrenaline and inhalation strategies in acute bronchiolitis. N Engl J Med. 2013;368(24):2286-93.

4. Rolfsjord LB, Skjerven HO, Bakkeheim E, Carlsen KH, Hunderi JO, Kvenshagen BK, et al. Children hospitalised with bronchiolitis in the first year of life have a lower quality of life nine months later. Acta paediatrica (Oslo, Norway : 1992). 2015;104(1):53-8.

5. Kunz B, Oranje AP, Labreze L, Stalder JF, Ring J, Taieb A. Clinical validation and guidelines for the SCORAD index: consensus report of the European Task Force on Atopic Dermatitis. Dermatology. 1997;195(1):10-9.

6. Skjerven HO, Rolfsjord LB, Berents TL, Engen H, Dizdarevic E, Midgaard C, et al. Allergic diseases and the effect of inhaled epinephrine in children with acute bronchiolitis: follow-up from the randomised, controlled, double-blind, Bronchiolitis ALL trial. Lancet Respir Med. 2015;3(9):702-8.

7. Pinto RA, Arredondo SM, Bono MR, Gaggero AA, Diaz PV. T helper 1/T helper 2 cytokine imbalance in respiratory syncytial virus infection is associated with increased endogenous plasma cortisol. Pediatrics. 2006;117(5):e878-86.

8. Buske-Kirschbaum A. Cortisol responses to stress in allergic children: interaction with the immune response. Neuroimmunomodulation. 2009;16(5):325-32.

9. Hu C, Li Z, Feng J, Tang Y, Qin L, Hu X, et al. Glucocorticoids Modulate Th1 and Th2 Responses in Asthmatic Mouse Models by Inhibition of Notch1 Signaling. International archives of allergy and immunology. 2018;175(1-2):44-52.
